# Is Fever a Conservative Force*Read at Meeting of Central Texas Medical Association, Waco, Texas, January 10, 1900.

**Published:** 1900-03

**Authors:** J. W. Embree

**Affiliations:** Belton, Texas


### TEXAS MEDICAL JOURNAL.

AUSTIN, TEXAS.

ESTABLISHED JULY, 1885.

PUBLISHED MONTHLY.—SUBSCRIPTION $1.00 A YEAR.

VOL. XV. AUSTIN, MARCH, 1900. No. 9.

### Original Contributions.

For Texas Medical Journal.

### Is Fever a Conservative Force.*

J. W. EMBREE, M. D., BELTON, TEXAS.

As a prelude I must insist, that when I discuss fever, I am a little off my hobby; for like most young, and some old physicians, obstetrics and surgery appeal to my consideration as specialties. Yet, by no means does the following discussion lack in interest to me—in fact I find that I wax warm—nor do I wish excitement to run so high among my hearers that you will suffer from the fever of fretting.

Poe speaks of "the fever called living," and this is the most prevalent of diseases, to say nothing of the fatality and contagion. It is a malady from which you and I are not exempt; but like *tout le monde,* we, too, are fellow sufferers with the moving millions. Of all the ills that flesh is heir to, fever and famine make inroads, whose unpitying tread leaves foot prints on the sands of time. 'Tis with you and me to allay these ravages, and let's to the task of alleviating enslaved humanity.

In our poor weak way we can only study cause and effect, while

*Read at Meeting of Central Texas Medical Association, Waco, Texas, January 10, 1900.

the great Designer holds the key to many mysteries whose infinitude baffles understanding.

I say with this proem; I come to talk with you of fever, which from the beginning of medicine has been recognized as an important symptom; has been studied through all the ages, and yet, today, we cannot say positively its cause, purpose and effect.

Hippocrates regarded high temperature of very much importance as a prognostic sign.

By the ancients, heat was regarded as an element of fire, and they compared the burning of fever to a consuming flame. The poet in speaking of it, says:

"Even through the bowels runs the scalding plague,
And wastes the flesh with floods of eddying fire."

Indeed, this question of the location of heat production has been variously decided.

Galen and Aristottle reckoned heat as centered in the heart, and the rate of pulse was an index to the amount of fever. As a diagnostic sign, they regarded the pulse of greater significance than the fever. Later on, body heat lost its prognostic value.

According to the theory and belief of Erasistratus, excessive heat was a morbid action in the vascular system, a forcing of the blood out of the veins into the arteries where it came in contact with the pneuma or vital spirit. They believed the pyramidal shape of the heart to be a special figure belonging to the flame. This was the belief until long after the discovery of the circulation.

When the chemical nature of respiration was discovered by Lavoisier, then the heart, as the center of heat production, was given up and heat was believed to be located in the lungs. Later on, all the organs of the body were believed to participate. So much for this brief reference, in outline of the fever theory as advanced primitively.

Today it is conceded that heat is a production of body metabolism and the equilibrium of heat production and elimination is controlled by special nerve centers located near the caudate nucleus. Furthermore, just as certain drugs acting on the pneumogastric increase or decrease the rapidity of the heart, in like manner may we not believe that there are similar influences which act on the heat centers in the brain to produce abnormal temperature.

When you poison an animal with curare, which acts by paralyzing the nerve endings in muscles, you will observe that the circulation and respiration continue at the normal rate; yet the temperature of the animal varies with the surrounding media. To me it seems but evident that the nervous system controls the heat mechanism.

The most general causes, and those we are to consider primarily, are the infective fevers. In these we have a specific micro-organism entering the system and fever resulting. Might we believe that this fever is due to the action of a toxine which they produce, acting on the heat centers? Could it not be that the height of the fever is according to the amount and rapidity of the toxic production? Furthermore, the duration varies according to the life history of the organism.

As an example, we will consider malaria. So long as the plasmodia remain in the red corpuscles, their poisonous production being pent up, the temperature remains normal. But reaching a certain stage they burst the corpuscle and then it is the fever rises, the toxine being liberated.

Anyhow, as a basis, we shall recognize fever as a symptom.

Ordinarily, it is understood as an elevation of the body temperature to some applicable thermal degree above normal. In broad terms we shall consider it to be at 100° F. and above, allowing for the wide diurnal range and whether axillary, oral or rectal record. Temperature being no guide as to the actual amount of heat produced or lost in any given time.

A temperature of 103° in some continued diseases as typhoid is of much more significance than a temperature of 105° in a disease of short duration. The length of time that it remains high is the prognostic point.

In mechanics, we recognize that if we want to raise the amount of energy, we must increase our fuel. So it is in our body economy. As fever begins to rise, there is a greater demand on metabolism, and since the digestive organs under fever are less active they are not able to supply the demand, so the reservoir of the body tissues must suffer the loss.

To understand the conservative process of fever, literally, we mean that it preserves the body in a safe or entire state, free from loss, waste, or injury. When we sit by the bedside of a typhoid patient who has suffered for weeks, and watch the constant drain on the system, we cannot but say that there is loss, waste, and injury.

But we want to know whether this fever is a necessary process for the cure of the disease, necessary for the destruction of the all-offending cause, the typhoid bacillus, if you please.

Leroy and Richter, in a series of experiments on the rabbit, found, by puncturing the corpus striatum, that they were able to induce an elevation of temperature. Then they would inoculate these with large doses of the virulent culture of diphtheria, pneumonia, and the like. They found that these animals were less susceptible to the disease, than normal animals treated in like manner. On this they based their conclusion, that fever and leucocytosis were necessary.

According to this, a tubercular patient suffering from daily temperature should be less liable to contract a disease than a healthy person. However, we know that their fatal termination is generally from some contracted disease, as pneumonia. The fever has so wasted the body and impaired the resistive powers, that such a patient is rendered more susceptible to all diseases.

As a means of diagnosis and prognosis, fever is nature's sentinel, warning us that there is a strife between the cells of the body and some foreign matter. Take an infected wound and watch the multiplication of the slumbering cells, the diapedesis of the leucocytes, and the battles that ensues. Nature brings to bear all her forces to prevent the entrance of the offending germs. In such a process, fever generally results; but it is due to the absorption of toxic substances and not that fever has risen to help in the process and to buoy the cells of the body to greater action.

There is no temperature at which the functions act better than at our normal temperature, and when elevated above this, there is destruction. They are less resistive to heat.

The range of a fever temperature cannot burn them out, for by growing the pathogenic micro-organisms, we find that fever is the temperature of their most productive growth. A sufficient degree of temperature to destroy the organism would in like manner destroy the cells of the body.

The higher the temperature, so great in proportion is the tissue waste, less active are the functions of elimination, upon which a greater amount of work is thrown.

Again, we cannot say whether fever or the toxic substance acting on the nervous system causes a rapid action of the heart. The pulse generally ranges about ten beats to every degree of elevation, and when very much out of proportion, as in yellow fever, it portends a grave condition. The rapid pulse is a means of forcing the heated blood from the deeper vessels to the periphery where the skin is made more active in the process of elimination, and where it can disperse its heat and return cooled, which is a better condition for the absorption of O and the giving off of $CO_2$.

Respiration, in like manner, is quickened and thus more heat is lost by warming the inspired air and volatizing the water with which it returns charged. Nature seems to exercise all her forces in the struggle to reduce and keep down the temperature. If fever were necessary, surely she would let it have its range and thus hasten the termination of the disease. In fever the heat-regulating mechanism is disturbed and fails to maintain the balance between heat production and dissipation. If the balance were maintained as in health, we would have a stable temperature at a higher level than the normal.

The destructive influence of fever is most marked on the protoplasm of the cells, being shown as cloudy swelling and fatty degeneration, according to the high degree of temperature. It will be observed to attack the muscular tissues, and accounts for so great weakness after a long spell of fever. Its effect on the heart is shown by the progressive loss in quality and power indicated by the heart becoming more frequent and less effective. The pulse should be considered a more valuable sign than the fever, both in diagnosing and prognosing a disease. As you know, the effect of a high temperature on the nervous system is shown by headache, sluggishness of mind, and finally delirium. Digestion is markedly impaired and this is regarded as a citadel in our treatment of all fever. Secretion and excretion are diminished. In fact, all the bodily functions suffer alike; the destructive influence of fever and the amount of loss will vary, according to how long it lasts and the height of its range.

Some may wish to say that there are many fevers; that by one attack we are granted partial or complete immunity. There are many theories as to how this immunity is obtained, such as the retention and exhaustion theories, but none will say that the fever grants the immunity. It is a result of the disease.

I am convinced, that if by some therapy or other means we were able to maintain the temperature at normal or thereabout, without injuring the heart by depressing drugs, we could render our patients more comfortable, all the functions would carry on their work better, the disease would be shortened, and the mortality lessened.

At present, our best results have been obtained from the judicious use of hydrotherapy. We have lessened the death rate of dreaded typhoid fever by its careful use. In pneumonia, I consider it of inestimable value. It acts as a stimulant to both respiratory and circulatory organs. You can watch a patient tossing and delirious, suffering the effects of a high fever; after using a cold bath and reducing the temperature, he will pass into a quiet sleep. It places a patient in the best condition to endure the disease.

If fever is a conservative process, we should try to increase it and thus hasten its termination. There are some diseases, the self-limited diseases that we are not able to check their course, but they must continue for a certain length of time; the life history of the organism must be run. Knowing that the tissue waste is in proportion to the high degree of temperature, I consider it as necessary to guard our temperature and keep it from ranging high, It is necessary to give a cold bath when the fever rises above 103°. Do not understand me as directing all attention to the treatment of the fever; it is but a symptom of the disease. But until we can treat the cause, it is one of our most important symptoms, and we treat most of our cases symptomatically.

When we can treat the cause as in diphtheria, then it is that we have true therapy. The discovery of the diphtheritic anti-toxine, I consider the greatest discovery in medicine of the century. It has rendered a very dangerous disease to be less dreaded than measles.

"Word may come tomorrow morning that some horrible disease
Has been mastered by some doctor, here at home or over seas;
They are stopping all the fevers and arresting all the ills
That the human form is heir to, with their serums and their pills;
They are causing men to wonder at their triumphs every day,
But people keep on dying in the same old-fashioned way!"

